# COVID-19 UK Lockdown Forecasts and R_*0*_

**DOI:** 10.3389/fpubh.2020.00256

**Published:** 2020-05-29

**Authors:** Greg Dropkin

**Affiliations:** Independent Researcher, Liverpool, United Kingdom

**Keywords:** COVID-19, UK, NHS, modelling, forecast, Bayesian, SEIR, R_0_

## Abstract

**Introduction:** The first reported UK case of COVID-19 occurred on 30 January 2020. A lockdown from 24 March was partially relaxed on 10 May. One model to forecast disease spread depends on clinical parameters and transmission rates. Output includes the basic reproduction number **R**_**0**_ and the log growth rate **r** in the exponential phase.

**Methods:** Office for National Statistics data on deaths in England and Wales is used to estimate **r**. A likelihood for the transmission parameters is defined from a gaussian density for **r** using the mean and standard error of the estimate. Parameter samples from the Metropolis-Hastings algorithm lead to an estimate and credible interval for **R**_**0**_ and forecasts for cases and deaths.

**Results:** The UK initial log growth rate is ***r*** = 0.254 with s.e. 0.004. **R**_**0**_ = 6.94 with 95% CI (6.52, 7.39). In a 12 week lockdown from 24 March with transmission parameters reduced throughout to 5% of their previous values, peaks of around 90,000 severely and 25,000 critically ill patients, and 44,000 cumulative deaths are expected by 16 June. With transmission rising from 5% in mid-April to reach 30%, 50,000 deaths and 475,000 active cases are expected in mid-June. Had such a lockdown begun on 17 March, around 30,000 (28,000, 32,000) fewer cumulative deaths would be expected by 9 June.

**Discussion:** The **R**_**0**_ estimate is compatible with some international estimates but over twice the value quoted by the UK government. An earlier lockdown could have saved many thousands of lives.

## Introduction

Although the first confirmed case of the novel coronavirus disease COVID-19 was identified on 30 January 2020, the UK government hesitated for some time whilst lab confirmed cases in England grew to 11,080 before a lockdown began on 24 March (1). The lockdown remains in place with no declared end date, but transport usage increased during April and moves to relax restrictions were signalled on 10 May.

In describing the spread of an infectious disease, one key parameter is the basic reproduction number **R**_**0**_, the expected number of individuals who will be infected by a single infectious person if the rest of the population is susceptible. If **R**_**0**_ > 1, the disease spreads, whilst if **R**_**0**_ < 1 it will die out. Recent preprints (2–4) and published articles (5, 6) have estimated **R**_**0**_ for COVID-19 internationally, but there is no clear consensus, and the true value may depend on social characteristics of the population.

As the disease progresses, patients who recover may acquire immunity, lessening the pool of susceptible individuals. However, at the initial stages almost everyone remains susceptible, and case numbers and deaths grow exponentially. A second key descriptive parameter is **r**, the rate of increase in log(cases) or log(deaths) during this exponential growth phase.

The disease spread can be approximated with a deterministic compartmental SEIR model (7) based on the numbers of patients who are Susceptible, Infected, Infectious (mild), Infectious (severe), Infectious (critical), Recovered, or Dead, and the rates of transition between these states. As a set of linked differential equations, the SEIR model can be integrated by numerical methods to forecast the future course of the disease.

Some of the transition rates in the model can be regarded as clinical parameters. For example, the rate at which people move from infected to infectious depends on the average length of the pre-infectious period, which is a clinical characteristic and might be similar in all populations. By contrast, the rate at which mildly ill people infect others depends on the pattern of social interaction in the community. These transmission parameters are likely to vary between countries.

UK government data underestimates the number of cases, as for some time there was no testing in the community, and the public was told not to inform the NHS if they felt only mildly ill. Until very recently, government data on COVID-19 deaths excluded deaths outside hospital. The most accurate available data on deaths is produced by the Office for National Statistics (ONS) using death registrations (8). The ONS coronavirus dataset covers England and Wales.

This paper begins by using the early ONS data to estimate **r** directly. The default clinical parameters in the SEIR model are then taken as fixed, and posterior samples of the transmission parameters are obtained, making use of the mean and standard error of the estimate for **r** and the fact that **r** is also an outcome of the model, conditional on the parameters. Using the model, these samples also generate samples for **R**_**0**_, giving an estimate and credible interval. The samples can be applied to forecast the spread of the disease in the context of a lockdown of specified effectiveness and duration, and to give credible intervals for the difference between forecasts in different lockdown scenarios.

## Materials and Methods

**Figure 1:**
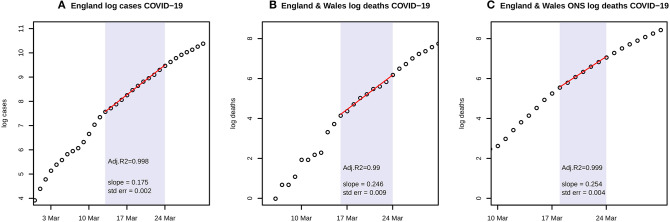
**(A)** shows the natural logarithms of UK government data for COVID-19 cases in England during March, with a linear fit to the portion from 13 to 24 March; **(B)** shows the corresponding graph for government data on deaths with a linear fit from 16 to 24 March; and **(C)** shows ONS data on deaths with a linear fit from 18 to 24 March. Each panel includes the estimate and standard error of the slope of the fitted line, and Adjusted *R*^2^.

A time series of confirmed COVID-19 cases in England and deaths in each UK nation is available from the UK Government (1). More complete data on deaths in England and Wales is available from the Office for National Statistics, using death registrations (8). Data on log(cases) and log(deaths) through to 31 March was examined for linearity, and a linear model M1 was then fitted to the appropriate portions before lockdown, as shown in Figure 1. Further analysis uses only the ONS data.

An unpublished implementation of the SEIR model, together with default parameters, clinical evidence, and **R** code is available online from Alison Hill (9, 10). The code includes functions Getr_SEIR and GetRo_SEIR to compute **r** and **R**_**0**_, respectively, depending on the parameters and population size. The **r** estimate uses the approximation that in the early period of growth, virtually the entire population is susceptible, and the SEIR model reduces to a linear differential equation with growth determined by the largest eigenvalue of its matrix. The model compartments all grow exponentially at this same rate during the early period, and their relative sizes are determined by the components of the corresponding eigenvector. The model is fitted by numerical integration using the **R** package “deSolve” (11).

The transmission parameters pertain to patients whose condition is mild, severe, or critical. Although the model also permits asymptomatic and pre-symptomatic transmission and seasonal variation, these options were not considered here. The three transmission parameters are estimated by an adaptive Metropolis-Hastings (M-H) algorithm, fixing all clinical parameters at the values chosen by Hill. A trio of scaled transmission parameters (b1^*^N, b2^*^N, b3^*^N) where *N* = Population, then determines **r** through Getr_SEIR. The likelihood of the trio is taken to be the gaussian likelihood for the resulting **r** value, with mean and sd given by the estimate and standard error of the growth coefficient in M1 fitted to the ONS data. A trio receives a higher or lower likelihood if the model gives a fitted log growth rate nearer or further from the empirical value obtained directly from the data. The prior for the trio is set as the product of independent gamma priors for each component, using the same shape and rate for each. Adaptive M-H was run (**R** package “MHadaptive”) (12) to generate a sample of length 50,000 after burn-in and thinning. Convergence was checked by standard diagnostic tests (**R** package “coda”) (13). The output of M-H is a posterior sample of the trio (b1^*^N, b2^*^N, b3^*^N). Since **R**_**0**_ is completely determined by the parameters, the rest of which are fixed, the sample generates a sample for **R**_**0**_. Highest Posterior Density Intervals (**R** package “HDInterval”) (14) were used as credible intervals. The code file paramest1.txt implements these calculations. All code files and data are available as Supplementary Material.

Forecasting cases and deaths uses the model and parameters and the assumed reduction in transmission as a result of the lockdown, but also requires an initial value for the numbers in each model compartment. As the projections are short term, Population was fixed at *N* = 66 million. The run was started from 18 March, the beginning of exponential growth in the ONS data. The unknown initial number *ini* of Infected cases on 18 March determines the numbers of Infectious (mild, severe, critical), Recovered, and Dead on that date as their ratios are determined by the eigenvector for the maximum eigenvalue. For a specified lockdown scenario, the model can be run forward from 18 March as a function of *ini*, giving a predicted value *E* of new deaths at each date, and a squared Pearson residual (*E*—*O*)^2^/*E* where *O* is the ONS new deaths for that date. To estimate *ini*, the summed squared Pearson residuals for a period in which *O* is known were minimised. The chosen period runs from 18 March through to 24 April, using the ONS data for deaths registered by 9 May (8).

**Figure 2:**
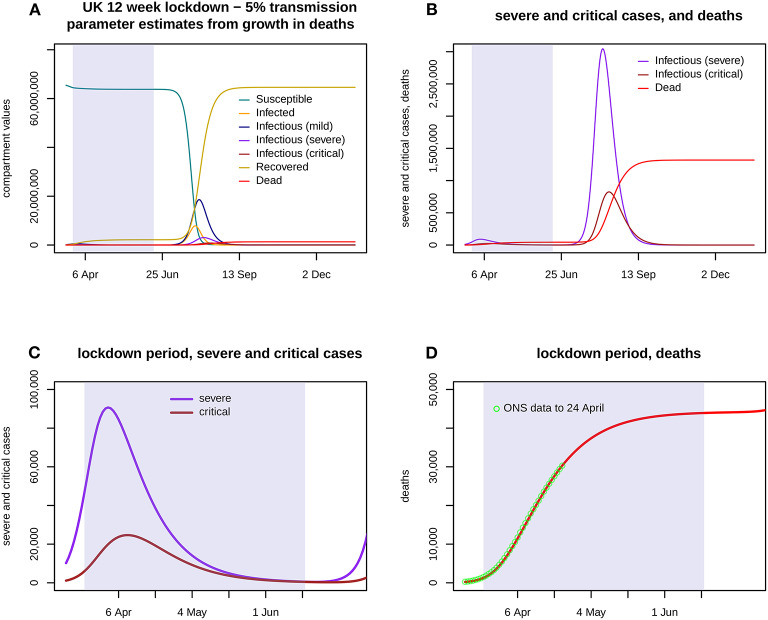
Assumes that a 12 week lockdown from 24 March holds all three transmission parameters (*b1*: mild, *b2*: severe, *b3*: critical) at 5% of their previous values with a run down from 14 March, with transmission fully restored from 16 June. **(A)** shows the first 300 days of the epidemic, with the lockdown portion shaded. The eruption of cases and deaths is delayed until after the lockdown ends. **(B)** shows severe and critical cases, and deaths, again for the entire 300 days. **(C)** shows severe and critical cases throughout the lockdown. **(D)** shows cumulative deaths during the lockdown, and the ONS data up to 24 April.

**Figure 3:**
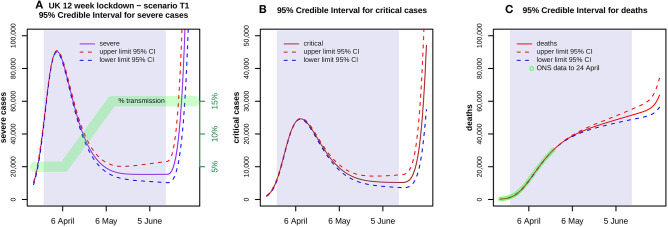
Shows 95% credible envelopes for **(A)** severe and **(B)** critical cases, and **(C)** deaths during a 12 week lockdown from 24 March with a transmission scenario T1 as indicated in **(A)** by the green band. ONS data up to 24 April is also shown in **(C)**. At each time point, the Highest Posterior Density 95% Intervals are found using 1,000 parameter samples. These indicate the uncertainty arising from transmission parameters whilst ignoring all other variability, whether in the choice of prior, the clinical parameters or the model itself.

Once the initial values on 18 March are estimated, the model can be run with the transmission parameters set at their mean values from the sample. Scenarios including a decrease in transmission prior to lockdown were chosen using the transport data in the Government daily briefings (15). One scenario, shown in Figure 2, assumed each *b*_*i*_ would be reduced to 5% of its sample mean throughout the lockdown. For Figure 3, with a scenario in which transmission is fixed at 5% for 2 weeks and then rises linearly to reach 15% by 11 May, 1,000 samples were drawn for (b1^*^N, b2^*^N, b3^*^N). The model was run using each sample to reset the initial conditions and forecast severe and critical cases and deaths during the lockdown. Highest Posterior Density credible intervals of mass 0.95 for the results at each time point then gave 95% credible envelopes.

The sensitivity of results was further tested by varying the choice of prior for the parameter sampling, or the Case Fatality Rate, or the start date of the 12 week lockdown.

**Figure 4:**
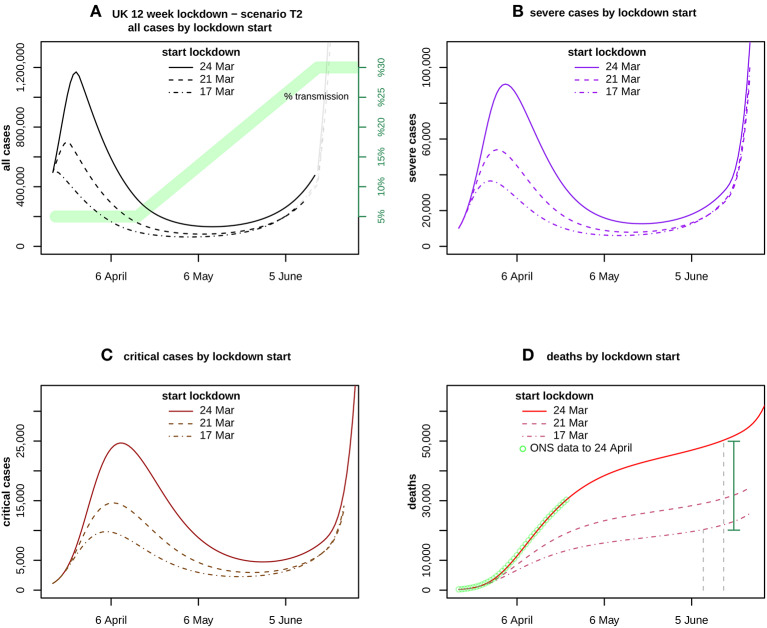
Assumes a transmission scenario T2 as indicated in **(A)** by the green band. It compares the forecasts for **(A)** all cases, **(B)** severe and **(C)** critical cases, and **(D)** deaths. ONS data up to 24 April is also shown in **(D)**. The curves compare the impact of lockdown start dates 24 March (solid), 21 March (dashed), and 17 March (dotdash). The vertical distance between the solid curve at 16 June and the dotdash curve at 9 June, is the estimated excess caused by delaying the lockdown for 1 week, shown by the green vertical bar.

In considering the impact of bringing forward the lockdown by a week, Figure 4 shows a scenario in which transmission is fixed at 5% for 3 weeks and then rises linearly to reach 30% by the end of 12 weeks, for lockdowns beginning on 24 and hypothetically on 21 and 17 March.

For a better understanding of the excess, deaths at the end of 12 weeks as sampled for Figure 3 were compared with deaths at the end of a 12 week lockdown beginning 17 March, using the identical choices for (b1^*^N, b2^*^N, b3^*^N), the same lockdown scenario, and the initial conditions as already estimated. The excess deaths due to a later lockdown are estimated by the mean and 95% CI for the pairwise difference of these two samples. The process was repeated using the lockdown scenario for Figure 4.

In the code file paramest1.txt, which generates the parameter samples and estimate and CI for **R**_**0**_, neither Getr_SEIR nor GetRo_SEIR depend directly on the parameter *u* governing the death risk for critical patients, but only on *u* + *g3* which is the reciprocal of the average length of stay in ICU (9, 10). Thus the estimates for **R**_**0**_ are independent of the Case Fatality Rate.

As this study uses only freely available anonymous data released by the UK government and Office for National Statistics, ethical approval was not sought.

## Results

Figure 1 shows log linear growth in the period just before the lockdown with all three data sources, and particularly with the ONS data in the period 18–24 March. A linear fit has Adjusted *R*^2^ = 0.999, and the coefficient of growth **r** = 0.254 with s.e. = 0.004. Adaptive M-H sampling passed the Raftery, Geweke, and Heidelberger tests for convergence. As discussed below, the gamma prior chosen for $b_{i}^{*}$N has shape = 1 and rate = 5. Posterior estimates of the transmission parameters, scaled by population *N* = 66 million, are b1^*^N = 1.095 with 95% highest posterior density credible interval (1.04, 1.15) whilst b2^*^N = 0.218 (0, 0.65) and b3^*^N = 0.202 (0, 0.6). **R**_**0**_ is estimated as 6.94 (6.52, 7.39).

Eight gamma priors with means ranging from 0.1 to 5 and coefficient of variation from 0.45 to 1.41 were specified by varying shape and rate parameters. The resulting estimates of **R**_**0**_ ranged from 6.8 to 11.06, with lower prior means giving lower **R**_**0**_ estimates. Priors with higher means also lead to higher estimates of deaths and excess deaths. With prior means in the range 0.1 to 0.5, **R**_**0**_ varied from 6.8 to 7.24. The chosen prior with mean = 0.2 and CV = 1 (shape = 1, rate = 5) was conservative, giving a comparatively low value of **R**_**0.**_

From the transport data (15) it appeared that transmission reduced after 14 March and reached a stable value around 26 March. Lockdown scenarios were considered with a 12 day run down to stable values of 10% and 5% of normal transmission.

The estimated number of infections on 18 March (see Methods re *ini*) was over 215,000 for the 10% lockdown, and over 245,000 for the 5% lockdown. Whilst the estimated *ini* gave a plausible fit to the ONS data with a 10% lockdown, the fit for a 5% lockdown was excellent, and all remaining scenarios assumed the lockdown began with transmission parameters at 5% of their previous values.

Figure 2 shows the results of a model run for a lockdown reducing each *b*_*i*_ to 5% of its estimate throughout a 12 week period 24 March−16 June, with a run down from 14 March. Severe cases peak in early April at over 90,000 but then decline slowly, critical cases peak in mid-April at nearly 25,000, and total deaths are nearly 44,000 by 16 June. Figure 3 shows the 95% confidence envelopes for the evolution of severe and critical cases and deaths during a lockdown which begins at 5% but rises linearly after 7 April to reach 15% on 11 May and then remains constant (scenario T1). The fit to the ONS data through to 24 April is still excellent.

## Sensitivity

These results are very sensitive to the starting date of the lockdown, as shown in Figure 4 which assumes 5% transmission for 3 weeks and then linear growth to reach 30% at the end of the 12 week lockdown (scenario T2). For T2 beginning on 24 March, severe cases peak in early April at over 90,000, critical cases peak in mid-April at nearly 25,000, and total deaths are nearly 50,000 by 16 June.

If the same scenario began 1 week earlier on 17 March, severe cases would peak at nearly 37,000 early in the lockdown, critical cases would peak at under 10,000 a week later, and deaths by the end of the 12 weeks would be under 22,000. These estimates are obtained by applying the mean values of the transmission parameter samples to each lockdown.

When the excess deaths due to delaying the start of the T2 lockdown from 17 to 24 March were sampled (see Methods), the mean value was 29,839 with 95% CI (28,037, 31,859). The corresponding result for a lockdown held at 5% throughout the 12 weeks was 26,783 (25,720, 27,781).

The **R**_**0**_ estimate and CI are independent of the Case Fatality Rate (see Methods). The results above and Figures were generated with CFR = 2%, the value shown by Hill (9). If CFR = 1%, a T2 lockdown beginning 24 March would result in peaks of around 181,000 severe and 49,000 critical cases, but deaths by 16 June would still total around 49,000. The rise in the predicted cases is due to an approximately doubled estimate of initial infections required to fit the observed deaths to 24 April.

**R**_**0**_ depends on the prior, but not excessively, as discussed above. The choice of prior has a stronger influence on the lockdown forecasts, but the estimates of excess deaths are less sensitive to this choice. Priors like the one chosen here, with lower mean values, lead to lower forecasts and lower estimates of excess death.

The various scenarios beginning at 5% give similar predictions for total deaths at the end of the lockdown period, and similar figures for the excess deaths caused by delaying its start. However, case numbers at the end of lockdown vary widely.

With 5% transmission throughout, only 3,426 active cases are predicted on 16 June, whilst 2,162,742 will have recovered and 43,895 died. Over the entire period including the initial estimates on 18 March, an estimated total of 2.15 m mild cases occur, of which 434,200 become severe and of those 109,863 then become critical.

With scenario T1, 159,338 active cases are predicted on 16 June, whilst 2,665,106 will have recovered and 51,162 died. Case totals over the period are an estimated 2.75 m mild cases, of which 538,088 become severe and of those 132,356 then become critical.

With scenario T2, 475,806 active cases are predicted on 16 June, whilst 2,699,764 will have recovered and 49,936 died. Over half of the active cases at the end of lockdown would be infected but not yet even mildly infectious. Case totals over the period are an estimated 2.9 m mild cases, of which 545,946 become severe and of those 131,057 then become critical.

## Discussion

This brief analysis uses an established deterministic SEIR model (9) for the development over time in the expected numbers of susceptible, infected, infectious, recovered cases and deaths, depending on parameters, some of which can be estimated from clinical studies since the outbreak of COVID-19. Other parameters concern the rate at which persons who have become infectious will infect others, depending on whether their clinical condition is mild, severe, or critical. Clinical parameters, such as the delay between “infected” and “infectious,” may not vary greatly between countries. By contrast, the rate at which infection is transmitted in the community depends on level and types of social interaction, which may vary over time in response to public policy, such as a lockdown, or weather and season. For hospitalised patients, transmission also depends on the level of protection for healthworkers and on environmental and infection control measures including cleaning and air quality. If the evolution of all these parameters were known, the model would predict the numbers of people in different stages of the disease, or death, over time. Of course the model itself may be inadequate, no matter how the parameters are chosen. The SEIR model is much simpler than the hierarchical model being developed in the recent Report 13 from Imperial College (16).

In the context of the SEIR model, in the early period of an epidemic, numbers of infected or infectious persons or deaths are all growing exponentially at the same rate, and so the slope of their logs is identical. The true numbers of COVID-19 cases in this period are unknown due to the lack of testing. The analysis here uses Office for National Statistics death registration data in England and Wales, whose daily numbers in March are close to twice the UK figures in Government briefings, and around 40% above the Government figures in the remaining period to 24 April. The UK government figures are also shown on the Johns Hopkins University COVID-19 dashboard (17).

I estimated transmission parameters for the UK, on the assumption that clinical parameters are fixed at the values already estimated by Hill. A likelihood was assigned to transmission parameters and samples obtained via the Metropolis—Hastings algorithm. These samples lead to an estimate and credible interval for the basic reproduction number **R**_**0.**_ In reality, the clinical parameters are not fixed and their estimates will develop with new research. Therefore, the samples may be biased by the assumed clinical values and may underestimate the variability of the transmission parameters, so the true CI for **R**_**0**_ in the UK may be wider.

The value found here is compatible with a recent analysis of global data by Sanche et al. (preprint (4)), who estimated **R**_**0**_ in the range 4.7–6.6, significantly higher than the value of 3.11 cited by the UK government (2, 18). It is also significantly higher than the European average value of 3.87 [3.01–4.66] estimated in Imperial College Report 13, which is based on hierarchical modelling of 11 countries (16) and will reflect “choice of serial interval distribution and the initial growth rate of observed deaths,” with some choices resulting in UK estimates in the range 4–6.

**R**_**0**_ itself is based on an idealised notion of perfect mixing, and the analysis here is pooled over the entire population, without stratification by age or any other characteristics. The model also assumes that length of stay in each compartment is exponentially distributed, but a recent article by Verity et al. (19) fitted gamma distributions to length of stay, and estimated the coefficient of variation at 0.35 which implies shape = 8, rather than shape = 1 (exponential). Gamma distributions with higher shape parameters are more tightly concentrated on their mean values, which would reduce the probability of patients remaining infectious for long periods. I have not adapted the model to allow for this.

The parameter samples also enable forecasts, which are not based on **R**_**0**_ but directly on the model and parameter estimates. The forecasts here exclude the possibility that asymptomatic or pre-symptomatic patients are infective, or that after recovery, individuals may again become susceptible.

Whilst the transmission parameter *b1* (mild) influences the spread in the wider community, *b2* (severe) and *b3* (critical) are key to the risk to healthworkers. The estimates for *b2* and *b3* are sensitive to the prior, because the likelihood is based on **r** which is less dependent on *b2* and *b3* than on *b1*, as 80% of cases are mild. Likewise **R**_**0**_ is much more sensitive to *b1* than to *b2* or *b3*, which narrows the CI for **R**_**0.**_ However, even if a prior is chosen to fix *b2* and *b3* at 0, the unrealistic limiting case, **R**_**0**_ is estimated at 6.71 (6.43, 7.00).

The various lockdown forecasts assume that *b1, b2*, and *b3* each initially reduce to 5% of their pre-lockdown values, a value chosen from the transport data published with the daily briefings from the government. The forecast depends on estimating the number of cases on 18 March, when exponential growth began. This estimate was chosen to optimise the fit to the ONS data for deaths registered by 9 May but which occurred by 24 April, as later data is likely to be revised upwards when registrations become available. With 5% initial transmission, the resulting curves do fit the available ONS data. The predicted curves for severe and critical cases throughout the lockdown would have overwhelmed the NHS, if all of these cases were admitted to hospital. On the other hand, as the ONS data also shows, many people are now dying in care homes or in their own homes, so not all severe or critical cases are admitted to hospital.

As the transport data also shows, bus services outside London continued at around 15% of pre-lockdown rates, and the lockdown began to weaken in mid-April. On 10 May, Prime Minister Johnson signalled that restrictions would be relaxed. Three scenarios, all beginning with 5% transmission, were chosen to represent possible dynamics. T2 provides for 3 weeks at 5% followed by a steady rise, reaching around 15% on 10 May and continuing to 30% by mid-June. Whilst all three scenarios result in similar numbers of predicted deaths by 16 June, they differ widely in the number of active cases at the end of the lockdown period. T2 appears plausible in England, though not elsewhere in the UK, and would result in 475,000 active cases in mid-June. Over half of these cases would not yet show clinical symptoms, and would be undetected without comprehensive testing. They represent a threat for the future course of the disease.

The forecasts from three scenarios show cumulative deaths between 44,000 and 51,000 by 16 June, more than double the expert prediction given on 25 March as the lockdown began (20). Speaking to the UK Select Committee on Science and Technology, and citing the Imperial College modelling report of 16 March (21), its principal author Prof. Neil Ferguson stated “fatalities would probably be unlikely to exceed about 20,000, with effectively a lockdown and an intense social distancing strategy, and it could be substantially lower than that.” However, as Prof. Ferguson also stated, “real-time analysis modelling, of the type we are doing now, will be needed to refine those precise estimates.”

ONS data (8) now shows over 35,000 COVID-19 deaths in England and Wales by 1 May, suggesting that the forecasts in this paper may be conservative. This analysis is based only on death certificates which mention COVID-19. It does not include excess deaths from other causes which may have arisen as NHS facilities were focused on the pandemic.

The forecasts are based on assumptions concerning transmission rates, which could be overturned by a systematic programme of testing, contact tracing, isolation and quarantine as advocated by the World Health Organisation (22). The UK ended contact tracing on 12 March, and is only now preparing to resume.

The delay in beginning a hypothetical 12 week lockdown has a strong effect on the outcome. If the T2 scenario began on 17 March rather than 24 March, deaths by the end of 12 weeks would fall by around 30,000 (28,000, 32,000). The other scenarios give similar results. The excess is due to the rapid increase of cases during the pre-lockdown period. It raises an unanswered question: why did the UK lockdown only start on 24 March?

## Data Availability Statement

Publicly available datasets were analysed for the study. These can be found here: UK Government (1); Office for National Statistics (8).

## Ethics Statement

Ethical review and approval was not required for the study on human participants in accordance with the local legislation and institutional requirements. Written informed consent for participation was not required for this study in accordance with the national legislation and the institutional requirements.

## Author Contributions

GD conceived of the study and carried out the analysis.

## Conflict of Interest

The author declares that the research was conducted in the absence of any commercial or financial relationships that could be construed as a potential conflict of interest.
