# Supplementary material for: COVID-19 UK Lockdown Forecasts and R0
Source: Front Public Health. 2020 May 29;8:256. doi: 10.3389/fpubh.2020.00256 (PMC7274132; doi:10.3389/fpubh.2020.00256)
Supplement: Supplementary file 1 [file Data_Sheet_1.PDF]

R Code files

All codes can be downloaded as the zip file Rcodes.zip

Fig1.txt produces Figure 1, using engcases.csv, ewdeaths.csv, and onsdeaths_regby9.5.csv

paramest1.txt applies adaptive Metropolis-Hastings posterior sampling ("MHadaptive") to sample transmission parameters using independent gamma priors on *b1*, *b2*, *b3* and a likelihood defined from a gaussian density for **r** using the mean and standard error of the estimate. Credible intervals use "HDInterval" and convergence is tested with "coda".

samoutf0be0s1r5.txt is an output file from paramest1.txt which can be used to avoid running paramest1.txt itself.

Fig2.txt produces Figure 2 by running the model with "deSolve", using the clinical parameters from Hill and the mean values of transmission parameters obtained from paramest1.txt, and assuming transmission is cut to 5% during a 12 week lockdown with a run down prior to lockdown.

Fig3.txt produces Figure 3 using 1000 samples of transmission parameters obtained from paramest1.txt and applying them with lockdown scenario T1. It then provides samples of excess deaths, using identical transmission parameter samples for lockdowns beginning 24 March and 17 March.

Fig4.txt produces Figure 4 using lockdown scenario T2, and shows the impact of varying the lockdown start date.
